# Factors associated with sickness certification of injured workers by General Practitioners in Victoria, Australia

**DOI:** 10.1186/s12889-016-2957-5

**Published:** 2016-04-06

**Authors:** Rasa Ruseckaite, Alex Collie, Maatje Scheepers, Bianca Brijnath, Agnieszka Kosny, Danielle Mazza

**Affiliations:** Institute for Safety, Compensation and Recovery Research, Monash University, 222 Excibition St, Melbourne, Victoria 3000 Australia; Department of Epidemiology and Preventive Medicine, School of Public Health and Preventive Medicine, Faculty of Medicine Nursing and Health Sciences, Monash University, The Alfred Hospital, Melbourne, Victoria 3004 Australia; Department of General Practice, School of Primary Care, Faculty of Medicine Nursing and Health Sciences, Monash University, Building 1, 270 Ferntree Gully Rd, Notting Hill, Victoria 3168 Australia; Monash Injury Outcomes Unit, Monash Injury Research Institute, Monash University, Building 70, Clayton, Victoria 3800 Australia; Institute for Work & Health, 481 University Avenue, Suite 800, Toronto, Ontario M5G 2E9 Canada; School of Public Health & Preventive Medicine, Monash University, The Alfred Centre, Alfred Hospital, 99 Commercial Road, Melbourne, VIC 3004 Australia

**Keywords:** General practice, Work injury, Certification, Return to work

## Abstract

**Background:**

Work-related injuries resulting in long-term sickness certification can have serious consequences for injured workers, their families, society, compensation schemes, employers and healthcare service providers. The aim of this study was to establish what factors potentially are associated with the type of sickness certification that General Practitioners (GPs) provide to injured workers following work-related injury in Victoria, Australia.

**Methods:**

This was a retrospective population-based cohort study was conducted for compensation claims lodged by adults from 2003 to 2010. A logistic regression analysis was performed to assess the impact of various factors on the likelihood that an injured worker would receive an alternate/modified duties (ALT, *n* = 28,174) vs. Unfit for work (UFW, *n* = 91,726) certificate from their GP.

**Results:**

A total of 119,900 claims were analysed. The majority of the injured workers were males, mostly age of 45-54 years. Nearly half of the workers (49.9 %) with UFW and 36.9 % with ALT certificates had musculoskeletal injuries. The multivariate regression analysis revealed that for most occupations older men (55-64 years) were less likely to receive an ALT certificate, (OR = 0.86, (95%CI, 0.81 – 0.91)). Workers suffering musculoskeletal injuries or occupational diseases were nearly twice or three times at higher odds of receiving an ALT certificate when compared to fractures. Being seen by a GP experienced with workers’ compensation increased the odds of receiving ALT certificate (OR = 1.16, (95%CI, 1.11 – 1.20)). Occupation and industry types were also important factors determining the type of certificate issued to the injured worker.

**Conclusions:**

This study suggests that specific groups of injured workers (i.e. older age, workers with mental health issues, in rural areas) are less likely to receive ALT certificates.

## Background

Work-related injuries and diseases can have serious consequences for injured workers, their families, society, compensation schemes, employers and healthcare service providers. Healthcare utilization and sick leave taken by injured workers create substantial costs for compensation schemes [1, 2]. Extended absence from work can also place injured workers and their families in a weaker financial position and increase social isolation [3, 4]. Unfortunately, long-term sickness absence is very high in many countries [5]; only about 50 % of those who are off work for more than 6 months return to their normal workplace duties [6, 7].

The importance of demographic, medical, economic, social and job-related factors influencing duration of disability and return to work (RTW) after illness has been examined previously [8–15]. For example, Heymans et al [9] showed that “moderate” or “poor” job satisfaction, higher pain intensity, and female gender predict longer work absence in workers suffering from lower back pain. Similarly, Oyeflaten et al [11] found that women, blue collar workers and those with previous long-term (mean 9.3 months, SD = 3.4) sick leave had a lower probability for RTW amongst workers with mental and musculoskeletal problems.

Although many studies have investigated factors that predict disability after work-related injuries, it is not yet known if the same factors determine the type of sickness certificate issued to injured workers by their General Practitioners (GPs). It is important to understand if these same factors apply to GP certification practices because GPs play a significant role in the RTW process in Australia, being the first point of contact with the healthcare system for many injured workers and the main “gatekeepers” to workers compensation and disability benefits [16].

In Australia injured workers are issued three types of certificates: unfit for work (UFW), alternate or modified duties (ALT) and fit for work [17]. A medical certificate should be original, contain the worker’s name, employer details, precise diagnosis, dates on which the examination took place and when it was issued, and also dates on which the worker was unfit [18]. If the worker is recommended ALT duties, the GP will then tick an appropriate box with opportunity for comment and further consultation outside the certificate itself.

Our recent analysis [16, 19] of administrative sickness certification data in the state of Victoria showed that the majority of workers receive UFW certificates, while only one third are certified as being able to RTW on alternate duties. To understand this discrepancy, we conducted a cohort analysis of administrative claims data to compare and contrast UFW versus ALT certificates. The aim of the present analysis was to establish whether demographic, occupational, industry, medical (GP caseload of injured workers), injury and socio-economic factors can be associated with the type of sickness certificate issued by a GP to a worker following a work-related injury or disease.

## Methods

### Study design and Settings

The state of Victoria in Australia had a working population of approximately 2.8 million as at June 2011 [20]. Employers in the state are required to maintain workers’ compensation insurance through the WorkSafe Victoria (WSV) unless they are able to self-insure, obtain insurance through the national workers’ compensation scheme, or if they are a sole trader. The WSV system provides coverage for approximately 85 % of the Victorian labour force. All injuries and illnesses that exceed the pecuniary threshold for healthcare expenses or have required more than 10 days work absence are required to be lodged with the WSV via one of six private insurers.

The Victorian workers compensation system requires production of a medical certificate in order to accept a compensation claim. Certificates can be submitted by GPs and physical therapists or by hospital-based medical practitioners. The medical certificate contains information that include the practitioner’s recommendation regarding fitness to work (UFW, ALT, fit for work) and the start and end date of the certificate [16]. There are statutory limits for the duration of UFW certificates defined in the state’s workers compensation regulations. Initial medical certification for a workers compensation claim can be of up to 14 days duration whilst subsequent certificates can be of up to 28 days duration.

This study was a retrospective population-based cohort study, for which the authors accessed the Compensation Research Database (CRD) established at the Institute for Safety Compensation and Recovery Research (ISCRR) at Monash University, Melbourne, Australia. The CRD contains de-identified case-level administrative data received from the WSV between years 1986-2012 [21, 22]. The CRD only contains details of sickness certificates issued for injuries sustained in the workplace, as periods of sick leave caused by pre-existing non-work related health problems are not recorded.

More detailed information on this dataset is provided elsewhere [16].

### Study sample

All data for accepted compensation claims lodged by working age adults (15 - 65 years) with a date of injury between 1 Jan 2003 and 31 Dec 2010 were extracted from the database (*n* = 217,076). Claims were excluded if:

- The claim was accepted prior to 2003, as there were no adequate data on sickness certificates available.
- The claim was for healthcare expenses only (i.e., the claim did not meet the 10 day work absence threshold, therefore no sickness certificate was issued) (*n* = 78,086, 35.6 %);
- The initial sickness certificate was written by a health practitioner other than a GP (*n* = 5439, 2.5 %);
- The information on duration of certificates contained logical errors, such as certification date prior to injury date and similar (*n* = 82, 0.04 %).
- Claims that had no sickness certificates associated with it (*n* = 9654, 4.4 %)
- Worker was issued a “fit for work” certificate or recommended a full RTW (*n* = 3915, 1.8 %). More specific and detailed inclusion/exclusion details are published elsewhere [16, 19].

In this study only the initial sickness certificates were included in the analysis, since in this database information recorded about subsequent certificates may be incorrect or missing. Sickness certificates of all individual claimants were organised into two pre-defined categories: (1) UFW certificates where GPs recommended a complete absence from work (*n* = 91,726) and (2) certificates where the GP recommended a RTW with ALT duties (*n* = 28,174).

Following several consultations within the research team, which included GPs, six categories of the most frequent worker conditions (injuries and diseases described by the Type of Occurrence Classification System (TOOCS) Third Edition (http://www.safeworkaustralia.gov.au/sites/SWA) to code injury and disease types) for issuing sickness certificates were included in the analysis: (1) fractures, (2) musculoskeletal diseases (MSD), (3) other traumatic injuries, (4) back pains and strains, (5) mental health conditions (MHC) including work-related stress and post-traumatic stress disorders, and (6) other diseases [16]. The TOOCS system is designed to code both injuries and diseases, and identifies the most serious injury or disease reported on the initial claim for workers’ compensation and allocates an appropriate code from the Nature of Injury/Disease Classification. If more than one injury or disease is reported, the most serious injury or disease that is likely to have the most adverse effect on the worker’s life is selected [16].

### Statistical analysis

Both univariate and multivariate logistic regression analysis was performed to assess the impact of a number of factors on the likelihood that an injured worker would receive an ALT certificate from their GP. In the present study, the model predicted ALT (i.e. ALT certificate was set as 1 and UFW as 0). The model consisted of demographic (age group, gender, residential location), occupational (occupation group and employer segment size), industry type, medical (GP caseload of injured workers), injury type and socio-economic factors each with two or more levels (see Table 1). Employer segment size is based on the employer’s annualised remuneration and is grouped into small - < $1 M, medium $1 M - $20 M, large - > $20 M and government.

**Table 1:** Risk factors investigated in the present study

Variable	Description
Age group	Age groups in 10 year age bands as per the Australian Bureau of Statistics (www.abs.gov.au);
Gender	Male/Female
Worker condition	Worker condition at the initiation of claim
Postcode	Local government area postcode transformed to the residential location: metro, rural, interstate, missing or unknown.
GP caseload	The GP caseload was calculated by adding the number of claims for each GP provider and dividing into four groups based on consultation with GP’s on what was considered low and high caseloads for a provider. Group 1 with 13 claims per provider (c/p) were considered low, group 2 with 14 – 26 c/p was low-medium, group 3 with 27 – 48 c/p was high-medium and group 4 with 49+ c/p was considered a high caseload (over the eight year period from 2003-2010).
Occupation group	The major occupation group for the claimant based on the Australian and New Zealand Standard Classification of Occupations (ANZSCO).
Employer segment size	This variable reflects the size of the employer where the injury took place. The segment size is classified into four groups determined by the organisation’s annual remuneration; <$1 M (Small); $1 M - $20 M (Medium); >$20 (Large); Government (Government).
Industry group	The major workplace industry group code based on the Australian and New Zealand Standard Industrial Classification (ANZSIC) 2006 codes.
Socio-economic Index (SEIFA)	The “Index of Relative Socio-economic Advantage and Disadvantage (IRSAD) - 2011 State Score”, refers to a classification by the Australian Bureau of Statistics that ranks areas in Australia according to relative socio-economic advantage and disadvantage based on information from the five-yearly Census. All areas are ordered from the lowest (10 % assigned 1) to the highest (10 % assigned 10) decile number. Each area is divided into 10 groups and assigned a decile number, each decile subsequently then have an equal number of areas not necessarily people

All factors had statistically significant contributions and were added to the multivariate model (Table 3). For the univariate analyses, all cases were included except for the Socio-Economic Indexes for Areas (SEIFA) [23] variable, which was missing for 241 cases. In the multivariate model these 241 (0.2 %) cases from the SEIFA variable were removed. The final sample for the multivariate model included 91,541 UFW cases and 28,118 ALT cases.

Cox and Snell [24] and Nagelkerke [25] pseudo R^2^ provides an indication of how well the fit of the model is relative to a ‘null’ model with no risk factors. The Nagelkerke R^2^ allows for the R^2^ to potentially reach 1.0, a correction to Cox and Snell that do not allow this [26].

All statistical analyses were conducted using the Statistical Package for Social Sciences (SPSS v.21). All statistical tests were conducted at the two-sided *p* < 0.05 level of significance. Study approval was obtained from Monash University Human Research Ethics Committee.

## Results

### General findings

A total of 119,900 claims with initial sickness certificates were included in this study. A descriptive summary of the variables is provided in Table 2 which outlines the number and proportion (%) of sickness certificates within each risk factor. The majority of the injured workers in both ALT and UFW categories were men, mostly between 45-54 years of age. Nearly half (49.9 %) of injured workers with UFW and 36.9 % of ALT certificates suffered from MSD. The most common occupation in the study sample was labourer, the most common industry – manufacturing, and the most common location of all injured workers was the metropolitan area of the state capital city.

**Table 2:** Profile of alternate duties and unfit for work certificates by category in Victoria, 2003-2010

	Alternate duties certificates		Unfit for work certificates		Total Certificates	
Factors	N	Row %	N	Row %	N	Row %
Total Claims	28,174	23.5	91,726	76.5	119,900	100
Age Group						
15 – 24 years	2827	22.4	9793	77.6	12,620	100
25 – 34 years	5551	24.7	16,956	75.3	22,507	100
35 – 44 years	7279	23.5	23,643	76.5	30,922	100
45 – 54 years	8307	23.7	26,714	76.3	35,021	100
55 – 64 years	4210	22.4	14,611	77.6	18,821	100
Gender						
Male	18,950	24.3	58,891	75.7	77,841	100
Female	9224	21.9	32,835	78.1	42,059	100
Worker Condition						
Fractures	2040	17.3	9756	82.7	11,796	100
MSD	14,062	29.3	33,884	70.7	47,946	100
Other traumatic injuries	3402	18.2	15,320	81.8	18,722	100
Back pains & strains	4200	21.0	15,765	79.0	19,965	100
MHC	608	4.9	11,871	95.1	12,479	100
Other diseases	3862	42.9	5130	57.1	8992	100
Local Government Area						
Metro	18,686	24.6	57,367	75.4	76,053	100
Rural	7214	20.9	27,233	79.1	34,447	100
Interstate	2274	24.2	7126	75.8	9400	100
GP caseload						
1 – 13 Claims/provider	6622	22.4	22,941	77.6	29,563	100
14 – 26 Claims/provider	6654	22.1	23,389	77.9	30,043	100
27 – 48 Claims/provider	6763	22.1	23,824	77.9	30,587	100
49 + Claims/provider	8135	27.4	21,572	72.6	29,707	100
Occupation						
Managers	1428	22.5	4909	77.5	6337	100
Professionals	2393	19.0	10,205	81.0	12,598	100
Technicians & trades	6473	24.7	19,688	75.3	26,161	100
Community & personal service	2792	17.1	13,532	82.9	16,324	100
Clerical & admin	978	21.4	3593	78.6	4571	100
Sales workers	926	23.6	2997	76.4	3923	100
Machinery operators & drivers	5625	26.7	15,463	73.3	21,088	100
Labourers	7559	26.2	21,339	73.8	28,898	100
Employer Segment Size						
Small	6190	19.3	25,916	80.7	32,106	100
Medium	12,576	25.4	36,876	74.6	49,452	100
Large	7851	28.6	19,570	71.4	27,421	100
Government	1557	14.3	9364	85.7	10,921	100
Industry						
Manufacturing	7733	31.0	17,232	69.0	24,965	100
Wholesale trade	2261	29.4	5442	70.6	7703	100
Mining	105	28.3	266	71.7	371	100
Electricity, gas, water & waste	296	26.8	808	73.2	1104	100
Professional scientific & technical services	581	25.4	1705	74.6	2286	100
Information media & telecommunications	207	24.8	628	75.2	835	100
Retail trade	1588	24.3	4957	75.7	6545	100
Transport, postal & warehousing	2166	22.5	7481	77.5	9647	100
Construction	2775	21.5	10,115	78.5	12,890	100
Administrative & support services	818	21.3	3027	78.7	3845	100
Rental hiring & real estate services	221	20.9	835	79.1	1056	100
Arts & recreation services	618	20.8	2356	79.2	2974	100
Accommodation & food services	765	20.3	3004	79.7	3769	100
Financial & insurance services	127	18.9	544	81.1	671	100
Healthcare & social assistance	3358	18.8	14,497	81.2	17,855	100
Education & training	1156	18.0	5261	82.0	6417	100
Agriculture, forestry & fishing	541	17.4	2577	82.6	3118	100
Public administration & safety	1297	16.7	6451	83.3	7748	100
Other services	11,561	25.6	4540	74.4	16,101	100
Socio-Economic Index						
Lowest 10 % (0-10 %)	3232	25.7	9359	74.3	12,591	100
Lowest 11-20 %	1626	21.1	6078	78.9	7704	100
Lowest 21-30 %	2064	23.5	6728	76.5	8792	100
Lowest 31-40 %	2853	22.9	9590	77.1	12,443	100
Lowest 41-50 %	2982	23.6	9658	76.4	12,640	100
Highest 51-60 %	3396	23.5	11,033	76.5	14,429	100
Highest 61-70 %	4208	24.5	12,998	75.5	17,206	100
Highest 71-80 %	2827	23.1	9402	76.9	12,229	100
Highest 81-90 %	3717	23.5	12,125	76.5	15,842	100
Highest 10 % (91-100 %)	1213	21.0	4570	79.0	5783	100

*MSD* musculoskeletal disorders, *MHC* mental health conditions

### Individual variable Univariate and Multivariate analysis

Table 3 summarizes the contributions of each risk factor in the univariate and multivariate model. Univariate analysis (step 1) for all nine category variables was conducted to identify significant individual predictors. The nine category variables were then added into the multivariate model (step 2).

**Table 3:** Odds ratio and significance of factors associated with the type of GP certificate being issued (Unfit for work vs. Alternate duties, where Alternate duties is the outcome)

	Univariate model		Multivariate model	
Factors	Odds Ratio	CI at 95 %	Odds Ratio	CI at 95 %
Age Group				
15 – 24 years [REF]	1		1	
25 – 34 years	1.13^a^	1.07 – 1.19	1.03	0.97 – 1.09
35 – 44 years	1.06	1.01 – 1.12	0.95	0.91 – 1.00
45 – 54 years	1.39^a^	1.02 – 1.13	0.96	0.91 – 1.01
55 – 64 years	1.32	0.94 – 1.05	0.86^a^	0.81 – 0.91
Gender				
Male [REF]	1		1	
Female	0.87^a^	0.84 – 0.89	1.06^a^	1.02 – 1.10
Worker Condition				
Fractures [REF]	1		1	
MSD	1.95^a^	1.88 – 2.09	1.89^a^	1.79 – 1.99
Other traumatic injuries	1.06	1.00 – 1.12	0.99	0.93 – 1.05
Back pains and strains	1.27^a^	1.20 – 1.35	1.19^a^	1.12 – 1.27
MHC	0.24^a^	0.22 – 0.26	0.25^a^	0.22 – 0.27
Other diseases	3.60^a^	3.37 – 3.83	3.32^a^	3.11 – 3.54
Local Government Area				
Metro [REF]	1		1	
Rural	0.81^a^	0.78 – 0.83	0.91^a^	0.87 – 0.94
Interstate	0.98	0.93 – 1.00	0.95	0.91 – 1.01
GP caseload				
1 – 13 Claims/provider [REF]^b^	1		1	
14 – 26 Claims/provider	0.98	0.94 – 1.02	0.93^a^	0.90 – 0.97
27 – 48 Claims/provider	0.98	0.94 – 1.02	0.89^a^	0.85 – 0.92
49 + Claims/provider	1.30^a^	1.25 – 1.35	1.16^a^	1.11 – 1.20
Occupation				
Managers [REF]	1		1	
Professionals	0.80^a^	0.74 – 0.86	0.83^a^	0.76 – 0.91
Technicians & trades	1.13^a^	1.05 – 1.21	0.91	0.84 – 0.97
Community & personal service	0.71^a^	0.66 – 0.76	0.80^a^	0.73 – 0.87
Clerical & admin	0.93	0.85 – 1.02	0.96	0.87 – 1.07
Sales workers	1.06	0.96 – 1.16	0.92	0.83 – 1.02
Machinery operators & drivers	1.25^a^	1.17 – 1.33	0.91	0.84 – 0.97
Labourers	1.21^a^	1.14 – 1.29	0.91	0.85 – 0.97
Employer Segment Size [REF]				
Small	1		1	
Medium	1.42^a^	1.38 – 1.47	1.38^a^	1.33 – 1.43
Large	1.68^a^	1.68 – 1.74	1.86^a^	1.78 – 1.94
Government	0.69^a^	0.65 – 0.73	1.24^a^	1.14 – 1.34
Industry				
Agriculture, forestry & fishing [REF]	1		1	
Mining	1.88^a^	1.47 –2.40	1.53^a^	1.18 – 1.97
Manufacturing	2.13^a^	1.94 – 2.35	1.54^a^	1.39 – 1.71
Electricity, gas, water & waste	1.74^a^	1.48 – 2.05	1.31^a^	1.11 – 1.55
Construction	1.30^a^	1.18 – 1.44	1.08	0.97 – 1.20
Wholesale trade	1.97^a^	1.78 – 2.19	1.49^a^	1.33 – 1.66
Retail trade	1.52^a^	1.36 – 1.70	1.21^a^	1.07 – 1.36
Accommodation & food services	1.21^a^	1.07 – 1.37	1.05	0.92 – 1.19
Transport, postal & warehousing	1.37^a^	1.24 – 1.53	1.06	0.95 – 1.19
Information media & telecommunications	1.57^a^	1.30 – 1.88	1.15	0.95 – 1.40
Financial & insurance services	1.11	0.89 – 1.97	0.93	0.73 – 1.17
Rental hiring & real estate services	1.26^a^	1.05 – 1.50	1.19	0.99 – 1.43
Professional scientific & technical services	1.62^a^	1.42 – 1.85	1.37^a^	1.19 – 1.58
Administrative & support services	1.28^a^	1.14 – 1.45	1.06	0.93 – 1.20
Public administration & safety	0.95	0.85 – 1.06	1.00	0.88 – 1.14
Education & training	1.04	0.93 – 1.17	1.12	0.98 – 1.27
Healthcare & social assistance	1.10	0.99 – 1.21	0.86	0.77 – 0.97
Arts & recreation services	1.24^a^	1.09 – 1.42	0.99	0.86 – 1.13
Other services	1.63^a^	1.46 – 1.82	1.34^a^	1.20 – 1.51
Socio-Economic Index				
Lowest 10 % (0-10 %) [REF]	1		1	
Lowest 11-20 %	0.77^a^	0.72 – 0.82	0.96	0.90 – 1.04
Lowest 21-30 %	0.88^a^	0.83 – 0.94	0.99	0.93 – 1.06
Lowest 31-40 %	0.86^a^	0.81 – 0.91	0.97	0.91 – 1.03
Lowest 41-50 %	0.89^a^	0.84 – 0.94	1.00	0.94 – 1.07
Highest 51-60 %	0.89^a^	0.84 – 0.94	0.98	0.93 – 1.04
Highest 61-70 %	0.93	0.88 – 0.98	1.04	0.98 – 1.10
Highest 71-80 %	0.87^a^	0.82 – 0.93	1.02	0.96 – 1.09
Highest 81-90 %	0.88^a^	0.84 – 0.93	1.05	0.98 – 1.11
Highest 10 % (91-100 %)	0.76^a^	0.71 – 0.82	0.95	0.88 – 1.03

*MSD* musculoskeletal disorders, *MHC* mental health conditions

^a^denotes *p* < 0.05

^b^per eight year period

The full multivariate model containing all nine category variables (inclusive of the variables within each category) was statistically significant, *X*^2^ (52, *N* = 120,186) = 8636.976, indicating ability to distinguish between injured workers who receive an ALT and UFW certificate. The model explained between 7 % (Cox and Snell R Square) and 10.5 % (Nagelkerke R Square) of the variance in certificate type.

Compared to younger workers aged 15-24, there was a significantly reduced likelihood of workers in the 55-64 age-category receiving an ALT certificate from their GP OR = 0.86 (by 14 %), (95%CI, 0.81 – 0.91). Compared to men, women were at a slightly increased (0.62 %) chance of being issued ALT certificates, OR = 1.06 (95%CI, 1.02 – 1.10).

Taking other variables into account, worker condition was a significant risk factor. Table 3 shows that workers with MSD, OR = 1.89, (95%CI 1.79 – 1.99), and other diseases, OR = 3.32, (95%CI, 3.11 – 3.54), were three times more likely to receive an ALT certificate than those with fractures, whereas workers with MHC, OR = 0.25, (95%CI, 0.22 – 0.27), were less likely to receive an ALT certificate than those with fractures, MSDs and other diseases.

Worker’s area of residence was also an important risk factor. Compared to workers from metropolitan areas, there was a significantly reduced likelihood of injured workers from a rural area, OR = 0.91, (95%CI, 0.87 – 0.94), and interstate, OR = 0.95, (95 % CI, 0.91-1.01) receiving an ALT certificate from their GP.

An analysis of GP caseloads showed that GPs with the highest case load (i.e. 49 and more claims per provider over the eight year period), OR = 1.16, (95%CI, 1.11 – 1.20), were more likely (by ~16 %) to issue an ALT certificate to an injured worker than those GPs who saw less than 13 injured workers over eight years.

In terms of worker occupation, compared to managers, only professionals, OR = 0.83, (95%CI, 0.76 – 0.91) and community and personal service workers, OR = 0.80, (95%CI, 0.73 – 0.87) were significantly less likely (by 17 and 20 %) to receive an ALT certificate.

Employer segment size was a significant risk factor associated with an ALT certificate. Workers from medium (OR = 1.38 by ~38 %), (95%CI, 1.33- 1.43), large (OR = 1.86 (by ~86 %), 95 % CI, 1.78-1.94) and government size organizations (OR = 1.24 (by ~24 %), 95 % CI, 1.14-1.34) were more likely to receive ALT certificates than those from small organizations.

When considering industry, injured workers from mining, OR = 1.53, (95%CI, 1.18 – 1.97), manufacturing, OR = 1.54, (95%CI, 1.39 – 1.71), wholesale trade, OR = 1.49, (95%CI, 1.33 – 1.66), professional scientific and technical services, OR = 1.37, (95%CI, 1.19 –1.58) and other not elsewhere classified industries, OR = 1.34, (95%CI, 1.20–1.51) were significantly more likely (by ~40 % - 50 %) to receive an ALT certificate compared to injured workers from the agriculture, forestry and fishing industry. Taking other variables into account, SEIFA was not associated with ALT certificate at all (Table 3).

## Discussion

### General findings

The results of the current study clearly indicate that older workers, those with MHCs and those living rurally are more likely to receive UFW certificates than workers with physical injuries, workers living in metropolitan areas and workers visiting GPs with a higher injured worker case load. The latter are more likely to receive an ALT certificate. It is yet unknown why certain factors are associated with ALT certificates; however assumptions can be made based on existing literature, which show that older workers are less likely to RTW because they may have childcare and family responsibilities, are closer to retirement and may recover more slowly from an injury because of age and other existing health issues [27–29]. Older workers (between the age of 55 and 64 years) also seem to have more difficulty adapting to modified duties [11, 30]. In contrast, younger adults have been shown to have more favourable employment outcomes after injury [4, 12, 31].

We also found that workers suffering from physical injuries and other diseases were more likely to receive ALT certificates than workers with MHCs. It could be that GPs are more inclined to recommend modified duties and earlier RTW to such workers with physical conditions because they are familiar with interventions and type of modified duties available at workplaces that would be appropriate for such conditions [32]. Moreover, there is still a stigma associated with MHC and health professionals may perceive injured workers with mental illness as having poorer health outcomes than they really have [16, 33]. Studies also show that when it comes to MHC claims GPs grapple with issues such as diagnostic uncertainty, conflicting medical opinions, poor communication between professionals and secondary concerns related to pain management, lack of motivation by the injured worker to RTW and sourcing appropriate care services [34–36]. It is also possible that accommodations for MHC are absent in workplaces and as such GPs may be reluctant to suggest a return to work.

In terms of occupation, manual workers are less likely to receive ALT certificates than managers. This suggests that working on alternate or restricted duty appears to be a viable option mainly in managerial positions, whereas manual labour occupations have been associated with more severe disabilities of longer duration, probably associated with UFW rather with modified duties [37, 38]. On the other hand, research also shows that occupation does not determine the type of sickness certificate [39], and that may be why the odds of receiving ALT certificate across other occupations are very similar (Table 3).

As opposed to the findings reported by Shiel et al [15], demonstrating that GP and general practice factors had no significant impact on likelihood of a ‘may be fit’ note being issued, we found that those workers who see GPs with a higher caseload of injured workers are more likely to receive ALT certificates. This suggests that GPs with higher caseload of injured workers are familiar with the workers’ compensation system, have a positive attitude towards RTW and modified tasks and therefore more likely to recommend ALT duties [40–42]. This finding also suggests that in order to achieve improved certification (i.e. higher proportion of ALT certificates) systems may want to steer injured workers towards more “experienced” GPs.

Employer segment size stood out as an important risk factor associated with ALT certificate. Injured workers from large enterprises were nearly twice as likely to receive ALT certificates as those who work for small size organizations. This corresponds to previous findings [12, 37] that showed working for larger companies was positively associated with return to work. Larger organisations are able to employ specialists in disability management [43], provide more information about modified duties and RTW and have greater flexibility in allowing workers to return to modified jobs [37]. Larger workplace size has been associated with a shorter duration of absence following a physical work injury because of an increased ability of larger workplaces to offer accommodations or alternate duties [44].

In terms of industry, workers from mining, manufacturing, electricity, gas, water and waste as well as wholesale trade industries are more likely (up to 50 %) to receive ALT certificates than workers from agriculture, forestry and fishing. Literature on industry as a predictor of RTW is scarce; however it is known that being a blue collar worker (i.e. performing manual labour) is associated with longer duration off work when compared to those workers who perform professional jobs [11]. While physically demanding occupations and employment in goods producing industries have been associated with slower RTW for physical injuries [45], studies on mental health claims have reported longer duration off work in government and educational industries compared to other industry sectors [46].

### Study limitations and strengths

To the best of our knowledge, this is the first study that explores the factors associated with the type of sickness certificate issued by a GP in Australia. In this study we were able to examine almost all the predicting factors previously reported in the literature.

There are several limitations to our analyses. First, in this study we analysed the initial sickness certificates only. Consequently, we could not ascertain for how long UFW certificates were issued and when (and/or if) the changeover to ALT certificates occurred, thus facilitating RTW. Second, we were unable to analyse other important factors, such as comorbidities, a previous history of sickness certification, expectations of sickness absence and motivation as this information was not available from the data collected. The opportunity to include these explanatory variables would have increased the robustness of the model. Finally, data from administrative datasets are subject to entry errors, miscoding and misclassification, which we could not control for.

### Practical applications

It is known that extended periods of sickness can negatively affect injured workers, their family, employers and lead to increased compensation schemes. Workers might have poorer health outcomes and require an increased number of health interventions, which are associated with higher compensation costs [47-49]. From a policy perspective, this study suggests that efforts to target specific groups of injured workers (i.e. older age, workers with MHCs in rural areas) and employers (e.g. smaller companies) could increase the awareness of benefits of modified and alternate duties and facilitate RTW for groups that are otherwise less likely to RTW.

## Conclusions

The findings of this study suggest that seeing a GP with a higher caseload of injured workers (Table 3) increases the odds of receiving an ALT certificate. Such GPs perhaps are more experienced and familiar with work related injuries and compensation schemes. Perhaps they are also aware of RTW benefits; therefore they recommend ALT duties more frequently. Ultimately, it will be necessary to target specific workers’ groups, where ALT duties might be implemented and; therefore, interventions will need to be trialled and modified. Further research and more rigorous study designs are needed to determine what interventions and practice guidelines would be mostly effective to improve GP sickness certification practices, RTW and health outcomes of injured workers.

### Ethics approval

Monash University Human Research Ethics Committee, Melbourne, Australia.

### Availability of data and materials

Access to the CRD is publicly available for researchers to use, under strict guidelines approved by the compensation authorities and the Monash University Human Research Ethics Committee. Information about the CRD data can be found at http://www.iscrr.com.au/evidence-data-and-research/using-data/compensation-research-database-crd. For further information on this database, or to request a data extract for research, please review the ISCRR data access policy and email CRD@iscrr.com.au.
